# Electrochemical Treatment of Industrial Wastewater Degrading Tetrabutylammonium Bromide Using a Quasidivided Cell Design

**DOI:** 10.1002/open.202500381

**Published:** 2025-10-27

**Authors:** Laura Lennartz, Tobias Stadtmüller, Sebastian Arndt, Patrik Stenner, Siegfried R. Waldvogel

**Affiliations:** ^1^ Evonik Operations GmbH Rodenbacher Chaussee 4 63457 Hanau Germany; ^2^ Karlsruhe Institute of Technology (KIT) Institute of Biological and Chemical Systems—Functional Molecular Systems (IBCS‐FMS) Kaiserstraße 12 76131 Karlsruhe Germany; ^3^ Department of Electrosynthesis Max‐Planck‐Institute for Chemical Energy Conversion Stiftstraße 34‐36 45470 Mülheim an der Ruhr Germany

**Keywords:** boron‐doped diamond, cold combustion, industrial wastewater, quasidivision, tetrabutylammonium

## Abstract

A robust, simple, and safe anodic treatment of an industrial wastewater is developed containing tetrabutylammonium (TBA) salts. The use of a quasidivided electrolysis cell set‐up proves to be the key to success. Quasidivision enables the generation of oxidizing mediators without the necessity of an expensive and/or fragile membrane as separator. Screening experiments with significantly different current densities between anode and cathode reveal a higher efficiency compared to similar current densities at both electrodes. Furthermore, acidification of the wastewater prior to electrolysis improves the degradation efficiency by prevention of sulfurous electrode coatings (electrofouling). Under optimized conditions, the concentration of TBA cations is diminished to levels (<1 ppm) far below those required by environmental guidelines. 99% of the TBA species are depleted in total with a degradation rate around 1 mmol TBA bromide/100 min with an energy consumption of 2.5 kWh L^−1^. The developed process is applicable to wastewater with a varying composition.

## Graphical abstract

An electrochemical treatment for real industrial wastewater containing tetrabutylammonium is established, targeting a reduction below 5 ppm. By utilizing the quasidivision approach, it provides a practical solution to the growing issue of alkylammonium‐containing wastewater, which is increasingly used in electrochemical synthesis.

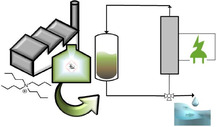

## 1 Introduction

Tetrabutylammonium bromide (TBABr) is a frequently used chemical in industrial processes and academic research. It serves as phase transfer catalyst, pH regulator, or as supporting electrolyte, among others.^[^ ^1^ ^]^ The advantage of superior stability even toward bacterial degradation compared to similar classes of substances, turns into a clear detriment when it comes to disposal and waste treatment.^[^ ^1b^ ^]^ Studies from 2018 indicate a persistent biocidal impact on water organism, and teratogenic effects towards the unborn child.^[^ ^1c^ ^]^

As a result, TBABr disposal is increasingly subject of environmental regulations restricting the use and requiring increasingly thorough removal thereof.^[^ ^1c^ ^]^ TBABr is classified as a Category 3 chronic aquatic chemical according to CLP classification and ECHA guidelines.^[^ ^2^ ^]^ Therefore, it should not be discharged into sewage systems without prior treatment.^[^ ^2^ ^]^ Treatment of wastewater is an important and contemporary topic in the chemical industry.^[^ ^3^ ^]^ A common method is the Fenton‐type oxidation process using iron catalysts and hydrogen peroxide.^[^ ^4^ ^]^ However, the emission of iron species into the environment is also legally regulated. Therefore, the generated iron sludge must be appropriately disposed of, resulting in a secondary off plant waste stream. Another often used approach is the incineration of the wastewater, which has an high energy demand rendering it uneconomical and unecological.^[^ ^5^ ^]^ A third and sophisticated approach is the removal of the targeted pollutants by membrane filtration.^[^ ^6^ ^]^ However, the stability of such membranes is limited by organic pollutants and the resulting concentrate has to be incinerated.^[^ ^6^ ^]^ Only the amount of waste that needs to be incinerated is minimized.

A modern method for wastewater treatment is the “cold combustion” by electrolysis.^[^ ^3^ ^b,^ ^3^ ^c,^ ^7^ ^]^ The general process has been known since 1889, but the use of electricity instead of chemicals has recently become attractive by the expansion of the renewable energy production.^[^ ^3^ ^b,^ ^8^ ^]^ The electrochemical wastewater treatment is based on the in situ generation of strongly oxidizing species at the anode that are capable of eliminating various organic pollutants in industrial wastewaters.^[^ ^3^ ^a,^ ^3^ ^b,^ ^9^ ^]^ The most efficient electrode material to date is boron‐doped diamond (BDD) due to its high overpotential for oxygen evolution and its preference to produce extremely reactive hydroxyl radicals in aqueous media.^[^ ^3a^ ^,^ ^3b^ ^,^ ^9^ ^,^ ^10^ ^]^ Hydroxyl radicals are capable of mineralizing organics up to CO_2_.^[^ ^3b^ ^,^ ^9a^ ^,^ ^10b^ ^]^ In addition, BDD is a sustainable choice compared to other commonly used anode materials, such as IrO_*x*_/RuO_*x*_ on titanium or PbO_2_, since it is fabricated from methane and poses no risk of leaching highly toxic heavy metals into solution.^[^ ^3b^ ^,^ ^9^ ^,^ ^10^ ^,^ ^10^ ^l,^ ^11^ ^]^ The effect of the cold combustion may be enhanced by adding inorganic chlorides or sulfur species, among others.^[^ ^9a^ ^,^ ^12^ ^]^ Chloride is oxidized to reactive (oxo)chlorine species (RCS) and sulfur to reactive (per)sulfate species (RSS).^[^ ^3b^ ^,^ ^9a^ ^,^ ^10b^ ^,^ ^13^ ^]^ Both are mediators with a high oxidizing power supporting the degradation of organic pollutants.^[^ ^3b^ ^,^ ^9a^ ^,^ ^10b^ ^,^ ^10j^ ^,^ ^14^ ^]^ Another effect of such additives is the improvement of the electrical conductivity. The applied voltage decreases versus the overpotentials for hydrogen and oxygen evolution reaction (HER, OER), which diminishes water splitting as the primary parasitic side reaction.^[^ ^3a^ ^]^ Consequently, higher current densities can be applied and allow a more compact electrolysis cell. An advantage of electrochemical treatment compared to conventional methods is the flexible adjustment of the degree of elimination of the targeted pollutant.^[^ ^3c^ ^]^ The correlation between reaction time and degradation level is predominantly mathematical in nature. While the reaction time is a significant factor influencing the degradation rate, it is not the sole variable that can be manipulated. The number of reactors employed also plays a crucial role. Given that electrochemical plants are frequently designed using a modular reactor system, increasing the number of modules can enhance the elimination rate as required. This approach allows for an improved degradation process without necessitating the complete construction of an additional facility. Levels below <1 ppm can be reached without alteration in the setup. If required, the pollution is eliminated beyond this amount by a complementary electrolysis cell with an adapted geometry; this is called water polishing. Amended environmental guidelines can be met by numbering up the electrolysis cells or by prolonged electrolysis time.

**Figure 1:**
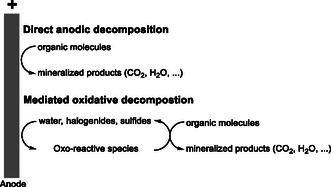
Oxidative degradation pathways of organic molecules at the anode (directly or mediated).^[^ ^8^ ^,^ ^15^ ^]^

In the following paragraphs, TBABr will be referred to as tetrabutylammonium cation (TBA), as our objective is to provide a treatment method applicable to the broader spectrum of TBA and tetraalkylammonium cations. Additionally, there are several established methods for lowering salt concentrations, including the remaining inorganic bromide‐containing salts, such as ion exchange processes. TBA or other tetraalkylammonium containing wastewaters accrue, when it cannot be substituted. Typically, such TBA concentrations are in the range of 4.000–12.000 ppm with impurities of sodium chloride and sulfide. Our ambition for an electrochemical treatment of wastewater was decreasing the TBA concentration below 5 ppm in agreement with concurrent environmental guidelines. We envisioned a simple, flexible, robust, and cost‐effective system, without the necessity for additional reagents. Importantly, the biodegradability of the treated wastewater was a central requirement for this approach. Our study aimed to propose a treatment option for TBA‐contaminated wastewater in general not only TBABr by testing the method on actual industrial effluent demonstrating its applicability in an industrial context (**Figure** **1**).

## 2 Results and Discussion

### 2.1 Materials and Methods

**Figure 2:**
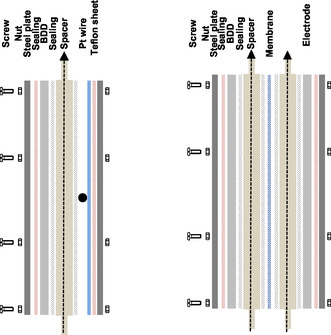
Cross‐section drawing of the undivided and the divided flow‐electrolysis cell set‐up (left, right) and frontal view (middle).

Batch‐type electrolysis trials were performed in a flow cell operated in cycling mode. The flow‐electrolysis cell had a plate‐electrode design and was tailor‐manufactured in our workshop on site. The sandwich of sealings in frame design (e.g., Viton, red), spacers (e.g., PP, brown), and electrodes (e.g., BDD, gray) and were pressed by two steel plates (e.g., dark gray), steel screws, and screw nuts at the edge of the plates (**Figure** **2**).

### 2.2 Experimental Results

Preliminary experiments were conducted at standard electrolytic conditions using electrolysis cells equipped with a planar BDD anode and a platinum cathode. The beaker or H‐cells were operated either undivided or divided using a cation‐exchange or an anion‐exchange membrane (CEM, AEM). The electrolysis conditions were varied in respect to current density, surface area difference, pH, and electrolysis time. However, in all examples no sufficient decrease or no decrease in TBA (=TBA+) concentration was detected via high performance liquid chromatography coupled to mass spectrometry (HPLC‐MS) confirming the outstanding stability of TBA towards oxidative degradation.

**Figure 3:**
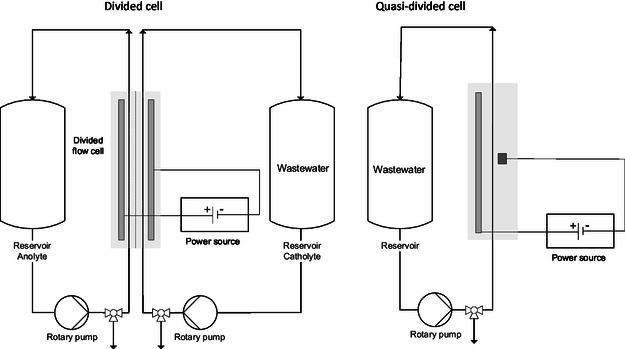
Flow scheme of divided flow cell (left) and a quasidivided flow cell (right) operated in cycling mode.^[^ ^16d^ ^]^

Interesting results were observed when experiments were conducted in a cycling batch‐type mode utilizing a divided flow cell (see **Figure** **3**). Only limited degradation of about 20% was observed when using the AEM setup (**Table** **1**, entry 2). When using Nafion 424 as a separator, the observation was made that the TBA permeated partially from the anolyte into the catholyte due to the positive charge. The remaining TBA in the anodic compartment was almost completely removed with only a residual concentration of 67 ppm left upon electrolysis (Table 1, entry 1). The TBA in the cathodic compartment remained unconverted. About half of the TBA was depleted in total (4640 ppm down to 2516 ppm). More experiments besides the listed in Table 1 conducted in the divided flow cell are listed in the Supporting Information.

**Table 1:** The catholyte was 0.1 M NaOH. In all experiments, BDD was the anode and tantalum supported platin sheet the cathode. Average terminal voltage range was 5–16 V. The current applied was 1.2 A.

No.	Membrane	Electrolysis time [min]	Anode area [cm^2^]	Cathode area [cm^2^]	Starting TBA concentration [ppm]	TBA concentration in anolyte [ppm]	TBA concentration in catholyte [ppm]	Total degradation [ppm]
1	CEM	290	80	15.6	4640	67	1453	2516 (54%)
2	AEM	405	80	15.6	5967	4885	23	1059 (18%)

Based on these preliminary results, the setup was changed to a quasidivided electrolysis cell to avoid the necessity of a membrane and the accumulation in the catholyte.^[^ ^16^ ^]^ Quasidivision means that the electrode surfaces differ strongly in area while operating in a undivided setup, which limits the mass transport kinetically at the smaller electrode, in our case the cathode.^[^ ^16a−d^ ^,^ ^17^ ^]^ The electrode reaction is thus mostly restricted to the bulk solvent.^[^ ^16a−d^ ^]^ The corresponding flow‐electrolysis setup was equipped with a BDD anode (active surface: 80 cm^2^) and a platinum cathode (employed surface of 15.6 cm^2^, tantalum supported) and was operated in cycling mode. The wastewater (300 mL) was kept in a reservoir and pumped through the flow electrolysis cell. The electrolysis was performed with a difference in surface area (Δ*s.a*) between anode and cathode of Δ*s.a* = 5.1:1 for 345 min. The use of a quasidivided setup affected the TBA degradation significantly. The experiment resulted in a total TBA degradation of 2825 ppm (44%) (**Table** **2**, Entry 1), in comparison to no degradation for the undivided experiment in batch‐type operation. Due to the obviated accumulation in the catholyte, we envisioned a complete oxidative degradation below 5 ppm with the quasidivided setup. With this result in hand, a series of screening experiments were performed on the difference in surface area between anode and cathode and the electrolysis time (Table 2, entry 2 + 3).

**Table 2:** Wastewaters were electrolyzed in a quasidivided setup with BDD as anode. Wastewaters were acidified with aqueous HCl to pH 2. The average terminal voltage ranged from 5 to 16  V.

No.	Pretreatment	Reaction time [min]	Cathode area	Anode area [cm^2^]	Δ*s.a.*	Δ*j *	Starting concentration [ppm]	Resulting concentration [ppm]	Total degradation [ppm]
1	None	345	15,6 cm^2^	80	5.1:1	5	6463	3638	2825 (44%)
2	None	360	6 cm^2^	80	13.3:1	13	6910	40	6870 (99%)
3	None	1060	3 mm^2^	80	2666:1	2600	6584	19	6565 (99%)
4	Acidification	710	3 mm^2^	80	2666:1	2600	5346	2	5344 (99%)
5	Acidification	395	3 mm^2^	80	2666:1	2600	5385	4	5381 (99%)
6	Acidification	615	3 mm^2^	80	2666:1	2600	5208	<1	5207 (99%)

**Figure 4:**
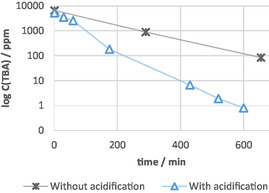
Anodic degradation versus time plot for acidified and non‐acidified wastewater. Experiments with the same electrolysis conditions were compared (Table **2**, Entry 3).

A beneficial effect was observed for higher differences in surface area, which was even more pronounced when going to smaller cathode surfaces (Table 2 Entry 2 + 3), in the range from 80 cm^2^ (anode) compared to 6 cm^2^–3 mm^2^ (cathode). A surface area difference, Δ*s.a* = 13.3:1, in surface area gave a lowering of the TBA concentration by about 99% from 6910 to 40 ppm (Table 2 Entry 2). With an even higher difference in surface area of Δ*s.a* = 2666:1 and a prolonged electrolysis time of 1060 min, the TBA concentration was reduced to 19 ppm (Table 2, Entry 3). However, we still not met our initial goal with an end TBA concentration of under 5 ppm. Throughout our experiments, we observed that sulfur contaminations in our wastewater disturbed the electrolysis by forming insulating coatings (electrofouling) at the anodic surface. For this reason, the effect of acidification as a pretreatment to electrolysis was examined. A low pH generally increases the oxidation potential in the electrolysis promoting the targeted degradation. The acidification of the wastewater prior to the electrolysis successfully removed the sulfur species by liberation as gaseous hydrogen sulfide. Three experiments were carried out subsequently using the optimized parameters, i.e., (Table 2: Entry 4–6) a very small cathodic surface (3 mm^2^) creating a significant difference in surface area of Δ*s.a*. = 2666:1 and prolonged electrolysis times from 395 to 710 min using acidified wastewaters. The targeted concentration of less than 5 ppm of TBA was achieved in all three experiments, reaching even a concentration below 1 ppm for TBA. The treatment efficiency was reproducible despite varying composition and homogeneity of the evaluated wastewater batches (Table 2: Entry 6). The beneficial effect of acidification on the current efficiency was clearly demonstrated by the rate of the degradation that was determined by taking samples over the course of electrolysis (**Figure** **4**). A three times faster degradation rate for the acidified wastewater (before pH = 8; after acidification pH = 2) was found compared to the nonacidified. The preclusion of sulfurous coating and a higher stability of the applied potential was observed. To understand TBA destruction mechanisms, we analyzed total organic carbon (TOC), organic acids, total nitrogen (TN), ammonia, nitrite, and nitrate levels before and after treatment as well as performing high resolution mass spectrometric (HRMS) analysis. We observed an 80% decrease in TOC but only a 30% reduction in organic acids, indicating carbon is primarily oxidized to carbonic acid. No increase in nitrate or nitrite suggests nitrogen from TBA remains in solution, while the decrease in TN implies nitrogen is released as gas. Additionally, ammonia levels increased, supporting the hypothesis of butyl chain oxidation and nitrogen elimination, resulting in ammonia release. Only traces (range of ppb) of chlorinated and or oxidized butyl chain derivates of the TBA were detected via HPLC‐HRMS. The analysis indicates that the butyl chains of the TBA are oxidized, while the nitrogen remains as ammonia. For further information look in the supporting information. Finally, the stability of the applied materials was evaluated. Alternative, less costly cathode materials than platinum or BDD were investigated with regard to their stability and performance. Stainless‐steel (1.4401) and graphite as cathode were unstable within the applied range of conditions. Thus, platinum remained the cathode material of choice.

## 3 Conclusion

An electrolyzer setup was developed and optimized for the cold combustion of TBABr containing wastewater. The cation TBA is a highly challenging substrate in terms of electrolysis as it is often used as relatively inert cation for supporting electrolytes. However, superior degradation efficiencies were reached by employing a quasidivided electrolysis cell and by prior acidification with hydrochloric acid. TBA concentrations were reproducibly depressed below 5 ppm, and even below 1 ppm, were achieved in agreement with environmental guidelines. The degradation rate corresponded to 1 mmol 100 min^−^^1^ specifically, 6 mmol TBA/615 min with an energy consumption of 2.5 kWh L^−^^1^. The results were well reproducible, and stable materials were identified.

Considerations should be directed toward addressing secondary components in the wastewater and the emitted exhaust gases. The emitted hydrogen sulfide from acidification can be captured using an activated carbon filter. The chlorine gas emitted during electrolysis can be converted into sodium chloride through the use of sodium hydroxide. This can then either be disposed, used as brine, or recycled into hydrochloric acid and sodium hydroxide through electrodialysis, thus allowing it to be recovered for the treatment process. The present study can be extended beyond cold combustion topic to organic electrosynthesis. In the future besides the feasibility of permeating Nafion 424 membranes, especially organic electrochemists, should be aware of the possible instability within high current densities as an supporting electrolyte.

## Conflict of Interest

The authors declare no conflict of interest.

## Supporting information

Supplementary Material

## Data Availability

The data that support the findings of this study are available in the supplementary material of this article.
