# Supplementary material for: Electrochemical Treatment of Industrial Wastewater Degrading Tetrabutylammonium Bromide Using a Quasidivided Cell Design
Source: ChemistryOpen. 2025 Oct 27;15(1):e202500381. doi: 10.1002/open.202500381 (PMC12835553; doi:10.1002/open.202500381)
Supplement: Supplementary file 1 — Supplementary Material [file OPEN-15-e202500381-s001.pdf]

ChemistryOpen

Supporting Information

Electrochemical treatment of industrial wastewater degrading tetrabutylammonium bromide using a quasi-divided cell design

Laura Lennartz, Tobias Stadtmüller, Sebastian Arndt, Patrik Stenner, and Siegfried R. Waldvogel*

Supporting information

Electrochemical treatment of industrial wastewater degrading tetrabutylammonium bromide using a quasi-divided cell design

Laura Lennartz, Tobias Stadtmüller, Sebastian Arndt, Patrik Stenner, Siegfried R Waldvogel*

Laura Lennartz, Tobias Stadtmüller, Dr. Sebastian Arndt, Patrik Stenner
Evonik Operations GmbH
Rodenbacher Chaussee 4, 63457 Hanau, Germany
Prof. Dr. Siegfried R Waldvogel
Karlsruhe Institute of Technology (KIT)
Institute of Biological and Chemical, Systems – Functional Molecula Systems (IBCS-FMS)
Kaiserstraße 12, 76131 Karlsruhe, Germany.
Prof. Dr. Siegfried R Waldvogel
Max-Planck-Institute for Chemical Energy Conversion
Department of Electrosynthesis
Stiftstraße 34-36, 45470, Mülheim an der Ruhr, Germany
E-mail:siegfried.waldvogel@cec.mpg.de
www.cec.mpg.de

1 **Contents**

2 General information S4
3 Materials and setup S6
3.1 Cell setup: schematic scheme S6
3.1.1 Cell setup 1: Divided flow cell S6
3.1.2 Cell setup 2: Quasi-divided flow cell S7
3.3 Constitution setup S7
4 General procedure S8
4.1 General procedure 1: divided flow setup S8
4.2 General procedure quasi divided flow setup S10
5 Pictures of the cell S12
5.1 Spacer S12
5.2 Stainless steel frame S12
6 Analysis S13
6.1. Carbon: TOC and organic acids S13
6.2. Nitrogen: TN, NH_4^+, NO_2^-, NO_3^- S13
6.3. ESI-HRMS data S13

2 General information

The wastewater was provided from Evonik industries and the major compounds were sodium chloride, sodium sulfide and tetrabutylammonium bromide in varying concentrations. All other chemicals were commercially available.

Chemicals

HCl: Hydrochloric acid fuming 37% for analysis provided by Merck

NaOH: Sodium hydroxide pellets for analysis provided by Merck

H_2SO_4: conc. Sulfuric acid for analysis provided by Merck

Electrodes

BDD electrodes were provided by Diaccon (Niob 2 mm with 12 ym diamond coating)

“Pt strips”: Pt on Ta welded; were provided by an in house workshop

Analysis

The LC Analysis were performed on an device from Agilent technologies (series 1260, quadrupole detector) with an RP 18 column. The quantitative analysis was done with an external calibration of tetrabutylammonium and determined via retention time and mass spectrometry.

We also tried GC-MS analysis, whether we detect any byproducts after treatment. However, we did not found any detectable compounds in the GC-MS.

For the cuvette tests following cuvettes from Hach were used:

Analysis	Cuvette type
**Organic acid**	LCK365
**Ammonia**	LCK305 and LCK 304
**Nitrite**	LCK341
**Nitrate**	APC339

For the analysis the Hach Lange Photometer DR3900 was used. The LT 200 from Lange was used for chemical breakdown prior analysis. For the TOC and TN analysis a total organic analyzer from Shimadzu was used.

Prior to measurement the samples were diluted with water.

Summarized interpretation

Since no significant concentrations of side products were detected in the initial samples, the analysis was conducted in SIM mode for TBA^+. However, in the quasi-divided optimized experiments, the samples were reanalyzed without SIM mode to verify the absence of oxidative side products. In all optimized experiments no detectable amounts of oxygenated or chlorinated forms of TBA were found.

Only one sample (Table S2 experiment No. 3) revealed the presence of chlorinated and oxygenated products of TBA. The detected amount were in all side products below 50 ppm. The ESI-HRMS spectra are attached in the document.

To gain a deeper understanding of the mechanism underlying TBA destruction, we analyzed the total organic carbon (TOC) and organic acid levels for carbon elimination, as well as total nitrogen (TN), ammonia, nitrite, and nitrate content before and after treatment. Our observations revealed an approximate 80% decrease in TOC, contrasted with only a 30% reduction in organic acids. This suggests that carbon is oxidized primarily into carbonic acid.

We found no increase in nitrate or nitrite levels, indicating that the nitrogen from the TBA molecule does not undergo oxidation and remains in solution. However, the observed decrease in TN suggests that nitrogen is emitted as a gaseous compound. Additionally, we noted an increase in ammonia levels. This combination of results, along with the relative increase in organic acids compared to TOC, supports the hypothesis of butyl chain oxidation and the elimination of nitrogen, resulting in ammonia being released.

Since no byproducts or oxidative degradation products of TBA were detected in the optimized experiments, we hypothesized the presence of gaseous oxidation products of TBA. Our proposed process implementation involves the thermal afterburning of gas emissions, a method commonly employed at chemical production facilities to convert gas emissions into energy. Given that hydrogen is invariably produced as a byproduct of electrochemical reactions in aqueous solutions, we considered utilizing this hydrogen while addressing the potential issue of hazardous oxidative byproducts associated with TBA. Consequently, we propose to combust our gas emissions in a thermal afterburner, thereby harnessing the generated hydrogen as an energy source. Additionally, the thermal afterburning system incorporates filtration mechanisms to manage pollutants such as nitrogen oxides (NOx) and carbon monoxide (CO). For the chlorine gas emission a gas scrubber with sodium hydroxide is utilized. The sodium chloride can then be upcycled again into HCl and NaOH. The HCl can be reused for acidification and the sodium hydroxide for the gas scrubber.

3 Materials and setup

3.1 Cell setup: schematic scheme

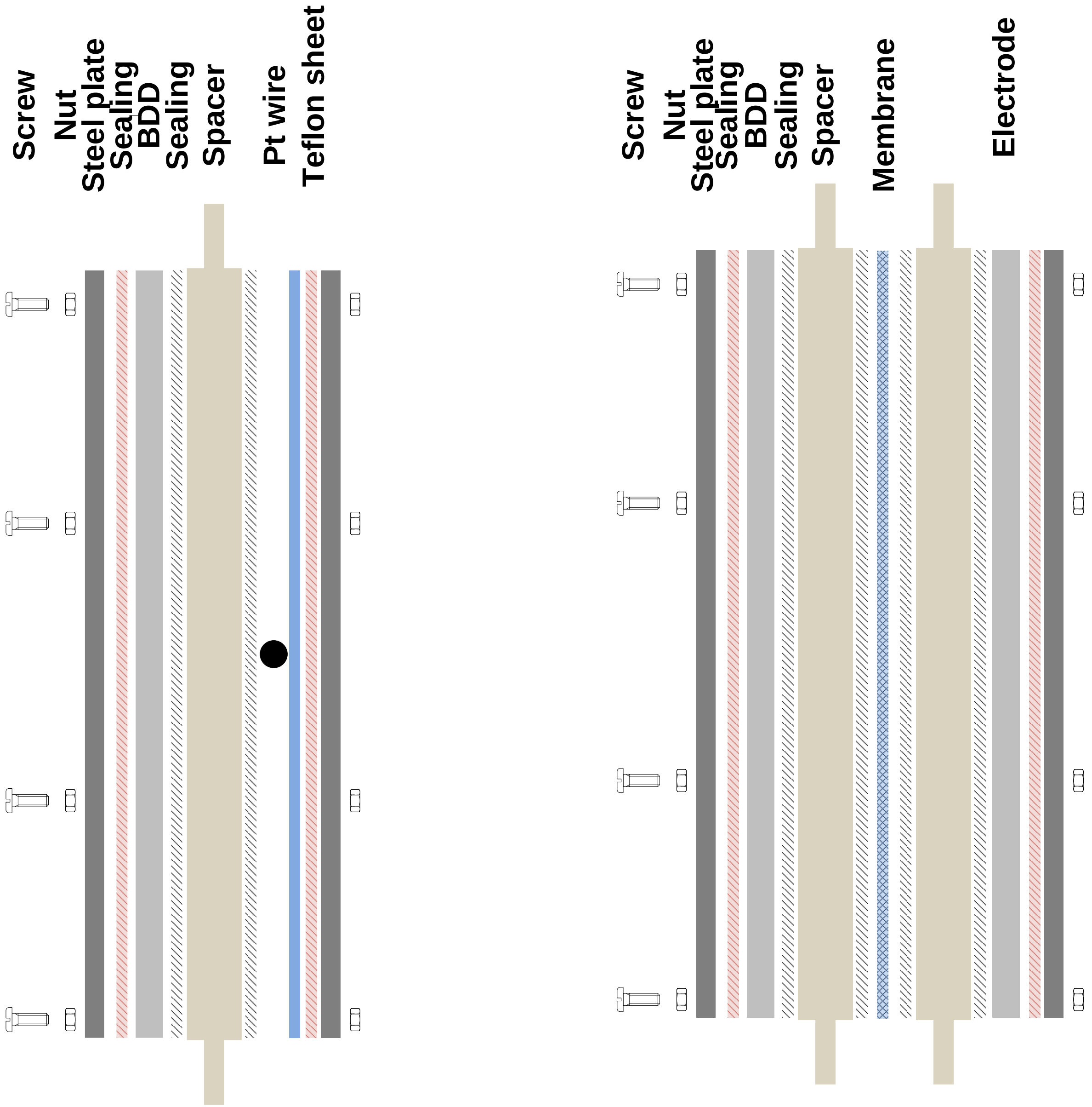

Figure S1: Schematic scheme of the cell setups.

3.1.1 Cell setup 1: Divided flow cell

The compartments of the cell are layered like a sandwich and are pressed together by two screwed steel plates. To isolate the electrodes from the steel plates a sealing (material: Viton, color: red) is used. On this sealing the electrode is placed. A small metal sheet (tantalum sheet with Pt strips) serves as a contact for the planar electrodes. Before and after the spacer (material: PP; color: brown) a sealing (material: EPDM or ePTFE; color: grey lines) is placed. Between the two sealings the membrane (color: blue) is fixated. Both types of membranes (material: Nafion 424 and AEM provided by Ralex) were inlayed a minimum of two days before use in water. After the membrane the sandwich replicates similar.

3.1.2 Cell setup 2: Quasi-divided flow cell

The same sandwich type flow cell is used, despite the double layered spacer and membrane is missing. An ePTFE sheet (color: blue) conduces as an inert back part of the cell and sealing, if a platin wire is used.

3.3 Constitution setup

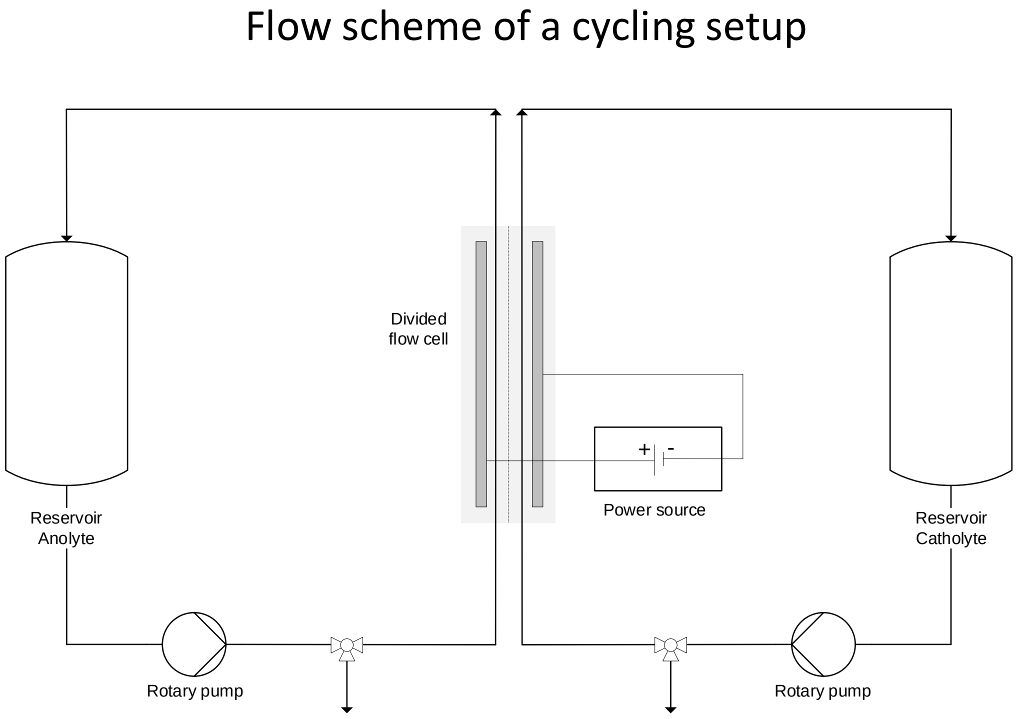

Figure S2: R&D scheme of the experimental setup.

For plumbing 6 mm teflon tubes were used. The rotary pump was a centrifugal pump provided by verder serie V-MD15. The three-way-valve was provided by EM-Technik (material: PP). As a reservoir an open glass vessel was used. The connection of the vessel and Teflon tube was provided from EM-Technik. The connection of rotary pump, Teflon tubing and also cell and Teflon tubeing, was a flexible EPDM tubing fixed by two hose clamps. The power source was provided from Rhode&Schwarz (R&S®HMP4000). As cables standard connectors were used and as clamps served standard clamps.

4 General procedure

4.1 General procedure 1: divided flow setup

The catholyte solution (300 mL of 3% H_2SO_4 or 0.1 M NaOH) was filled in the catholyte reservoir. In the anolyte reservoir the provided industrial wastewater (300 mL) was filled. After starting the rotary pumps, the fluid level on the vessel was marked. If the fluid level got below this level during electrolysis, the difference was refilled with distilled water. After a short time pumping the solution through the chamber, the electrolysis was started. While electrolysis the voltage was checked regularly and noted. In some experiments samples were taken while the experiment in an amount of 2 mL. The volume of both solutions was checked after electrolysis, either by volumetric control or weighting. After every experiment the setup was cleaned by flushing with water and ethanol serval times.

No	Pretreatment	time / min	Anode	cathode	anode area / cm^2	cathode area / cm^2	applied current	Current density anode A/m2	Average voltage / V	Membrane	anolyte	catholyte	Result catholyte /ppm	Result Anolyte/ ppm	Starting ppm	Difference
**1**	none	315	BDD	Steel	80	80	1.2 A	150	15	Nafion 424	Wastewater	3% H_2SO_4	8033	102	~10000-12000	
**2**	none	355	BDD	Steel	80	80	5.0 A --> 1.0 A in 5 steps	625 --> 125	12	Nafion 424	Wastewater	3% H_2SO_4	12384	74	~10000-12000	
**3**	none	905	BDD	Steel	80	80	5.0 A	625	12	Nafion 424	Wastewater	3% H_2SO_4	9988	10	~10000-12000	
**4**	none	535	BDD	Steel	80	80	5.0 A --> 1.2 A drop after 2 h	625 --> 150	12	Nafion 424	Wastewater	3% H_2SO_4	7705	88	10000-12000	
**5**	none	290	BDD	Steel	80	80	1.2 A	150	10	Nafion 424	Wastewater	0.1 M NaOH	1076	4348	6744	1320
**6**	add NaOH to anolyte before	395	BDD	Steel	80	80	1.2 A	150	9	Nafion 424	Wastewater	0.1 M NaOH	1076	3817	6325	1432
**7**	add NaOH to anolyte before	345	BDD	Steel	80	80	5.0 A	625	15	Nafion 424	Wastewater	0.1 M NaOH	4397	52	4941	492
**8**	none	260	BDD	Platin strips	80	15,6	5.0 A	625	13	Nafion 424	Wastewater	0.1 M NaOH	782	576	6287	4929
**9**	none	775	BDD	Platin strips	80	15,6	5.0 A	626	13	Nafion 424	Wastewater	0.1 M NaOH	1883	1	6728	4844
**10**	none	290	BDD	Platin strips	80	15,6	1.2 A	150	12	Nafion 424	Wastewater	0.1 M NaOH	1453	671	4640	2516
**11**	none	400	BDD	Platin strips	80	15,6	1.2 A	150	7	AEM	Wastewater	0.1 M NaOH	41	5180		-5221
**12**	none	405	BDD	Platin strips	80	15,6	1.2 A	150	7	AEM	Wastewater	0.1 M NaOH	23	4885	5967	1059

Table S1: All divided experiments are listed.

4.2 General procedure quasi divided flow setup

The industrial wastewater (300 mL) was filled into the reservoir and the rotary pump was started. After starting the rotary pumps, the fluid level on the vessel was marked. If the fluid level got below this level during electrolysis, the difference was refiled with distilled water. After a short time pumping the solution through the chamber, the electrolysis was started. While electrolysis the voltage was checked regularly and noted. In some experiments samples were taken while the experiment in an amount of 2 mL. The volume of both solutions was checked after electrolysis, either by volumetric control or weighting. After every experiment the setup was cleaned by flushing with water and ethanol serval times.

Table S2: All undivided (quasi divided) experiments are listed. The listed current density are the current densities of the anode as the working electrode.

No	Pretreatment	Time / min	Anode	Cathode	Anode area / cm^2	Cathode area	Applied current	delta s.a	Current density anode A/m^2	Average voltage / V	Result / ppm	Starting ppm	Difference
**1**	none	345	BDD	Pt strips	80	15.6 cm^2	2.0 A	5.1:1	250	5,5	3638	6463	2825
**2**	none	50	BDD	Pt strips	80	6 cm^2	5.0 A	5.1:1	625	7,7	5744	6827	1083
**3**	none	360	BDD	Pt strips	80	6 cm^2	5.0 A	13.3:1	625	10	40	6910	6870
**4**	none	1065	BDD	Pt wire	80	3 mm^2	0.5 A	13.3:1	62.5	5	2778	6440	3662
**5**	none	1060	BDD	Pt wire	80	3 mm^2	5.0 A	13.3:1	625	11	19	6584	6565
**6**	none	260	BDD	Graphite	80	1 cm^2	5.0 A	80:1	625	16	323	7185	6862
**7**	acidification with HCl	710	BDD	Pt wire	80	3 mm^2	5.0 A	2666:1	625	11	2	5346	5344
**8**	acidification with HCl	395	BDD	Pt wire	80	3 mm^2	5.0 A	2666:1	625	12	4	5385	5381
**9**	acidification with HCl	615	BDD	Pt wire	80	3 mm^2	5.0 A	2666:1	625	12	1	5208	5207
**10**	acidification with HCl	850	BDD	BDD	80	1 cm^2	5.0 A --> 1.0 A	80:1	625 --> 125	10	286	6318	6032
**11**	acidification with HCl	850	BDD	BDD	80	1 cm^2	5.0 A --> 1.0 A	80:1	625 --> 125	11	117	6296	6179

5 Pictures of the cell

5.1 Spacer

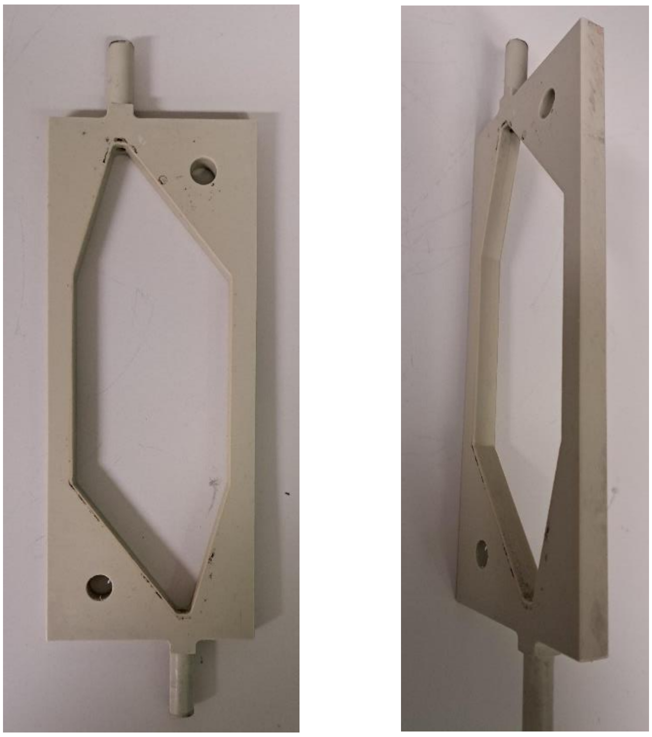

Figure S3: Front view (left) and side view (right) of the spacer. In and outlets are at the top and the bottom and connected to the pump and vessel; outer dimension 20 cm x 8 cm.

5.2 Stainless steel frame

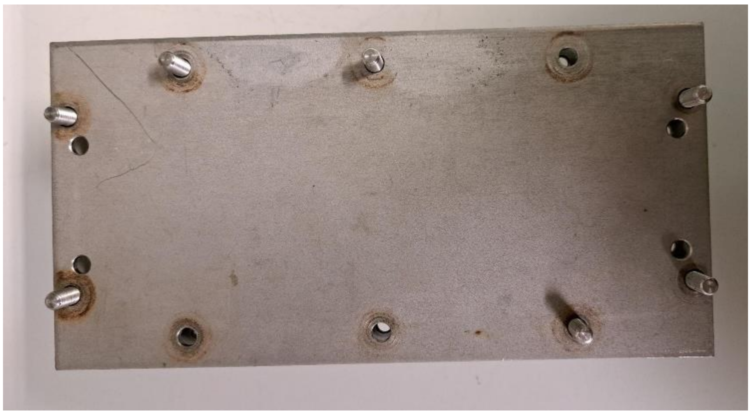

Figure S4: Stainless steel frame as backbone for the sandwich setup. Holes are for screws and nuts to tighten the setup; outer dimension 22 cm x 10 cm.

6 Analysis

To investigate the mechanism of the mineralization of the tetrabutylammonium further, analyses were performed. Besides the reduction of TBA as such, analyses were performed to watch out for species which could be harmful to the environment.

6.1. Carbon: TOC and organic acids

To determine whether only the TBA content decreases or if there is also a reduction in the total organic content (TOC), an analysis of our Experiment No. 7 from Table S2 was conducted. The results are shown in This description is primarily a well-informed hypothesis, as organic acids are known to undergo reactions during electrolysis, such as in the Kolbe reaction, leading to the formation of carbon dioxide. Consequently, these acids are more likely to react and diminish in concentration during our experiment compared to other carbon structures. However, it is important to note that this remains an assumption.

Table S3. There is a total reduction in the total organic content (TOC) from 20953 mg/L to 4027 mg/L, representing an elimination rate of 80%. This result suggests that not all carbon compounds are converted to carbon dioxide or other gaseous forms. To explore whether the mechanism for the degradation of the butyl chains involves the oxidation of the alkane chains to form organic acids, we also measured the content of organic acids. The results indicate a decrease from 2292 mg/L to 1621 mg/L, reflecting a 30% elimination rate compared to the 80% observed in the TOC. This suggests a degradation mechanism in which the alkane chains are oxidized to organic acids. This finding is promising, as organic acids are generally more biologically degradable than alkane chains. However, there is no certainty that the organic acids detected in the sample at the conclusion of the experiment are not merely the residual organic acids present from the initial conditions. This description is primarily a well-informed hypothesis, as organic acids are known to undergo reactions during electrolysis, such as in the Kolbe reaction, leading to the formation of carbon dioxide. Consequently, these acids are more likely to react and diminish in concentration during our experiment compared to other carbon structures. However, it is important to note that this remains an assumption.

Table S3: Measurements of TOC and organic acids before and after treatment.

Sample	TOC in mg/L	Organic Acids in mg/L
**Prior treatment**	20953	2292
**After treatment**	4027	1621

6.2. Nitrogen: TN, NH_4^+, NO_2^-, NO_3^-

To investigate the fate of nitrogen, given that TBA is a quaternary ammonium salt, we measured the same sample for total nitrogen (TN), ammonia, nitrate, and nitrite. The results indicate a total reduction in TN from 287 mg/L to 153 mg/L, reflecting an elimination rate of approximately 50%. This suggests that about 50% of the nitrogen content is emitted during the experiment.

To determine whether nitrogen is released as NOx gases, ammonia, or other forms, we also analyzed ammonia, nitrate, and nitrite levels. While we were unable to conduct an inline gas analysis due to limitations in our laboratory's analytical capabilities, we did not observe any significant increase in nitrite or nitrate after treatment. This finding suggests that a high amount of nitrous gases is not being emitted, which is beneficial for wastewater treatment, as nitrate and nitrite are hazardous compounds in water. The ammonia content increased from 0.5 mg/L to 2.9 mg/L. Although this is a modest increase, it provides insight into the degradation mechanism of the TBA molecule. It indicates that the butyl chain is oxidized to organic acids, while ammonia remains as the nitrogen byproduct, suggesting that carbon may be oxidized first in the process.

Table S4: Measurements of TN, NH_4^+, NO_2^-, NO_3^- before and after treatment.

Sample	TN in mg/L	NH_4^+ mg/L	NO_2^- mg/L	NO_3^- mg/L
**Prior treatment**	287	0,5	0,05	3,2
**After treatment**	153	2,9	0,08	3,4

6.3. ESI-HRMS data

Here are the spectra of the detected side products of experiment No.3 of table S2 attached. The range of concentration is in all cases below 50 ppm. The mass 276,24525 and 310,20628 were also detected in normal HPLC-MS measurements. All other masses were not detectable without high resolution mass spectrometry indicating a concentration range in ppb for these side products. In all optimized quasi divided experiments no side products were detected via standard HPLC-MS.

The substance of Figure S6 with the composition $C_{16}H_{36}N_1O_1$ indicates a mono-oxidized form of a intact tetrabutylammonium species. The substances of Figure S7, Figure S9, Figure S11 and Figure S13 with the composition $C_{16}H_{36}N_1Cl_x$, with x=1,2,3 or 4 reveals, that and intact tetrabutylammonium besides oxidation, also gets chlorinated. The substances detected in Figure S8, Figure S10, Figure S12 and Figure S14 with the composition of $C_{16}H_{36}N_1O_1Cl_x$, with x=1,2,3 or 4 shows a intact tetrabutylammonium with a single oxidation and mono or multiple chlorination's. This indicate, that the oxidation and chlorination of the tetrabutylammonium is not selective. The chlorination reaction may result due to the high sodium chloride concentration in the wastewater and radical chlorination reactions. The isotopic ratio appears to be normal, specifically 3:1 for 35Cl to 37Cl.

The majority of substances, apart from those shown in Figure S7 and Figure S11, were not detectable through standard HPLC-MS analysis and were identified only in this unoptimized experiment. Therefore, we assert that these minor traces do not pose a significant environmental impact. However, if such a wastewater treatment is implemented, it is essential to monitor these traces and discuss them with the environmental department.

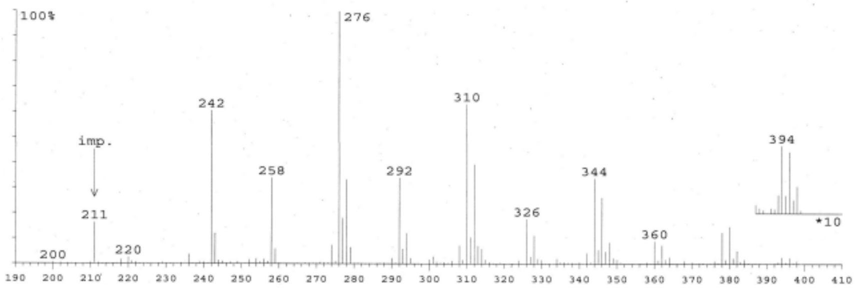

Figure S5: Mass spectra of the HR-ESI-MS.

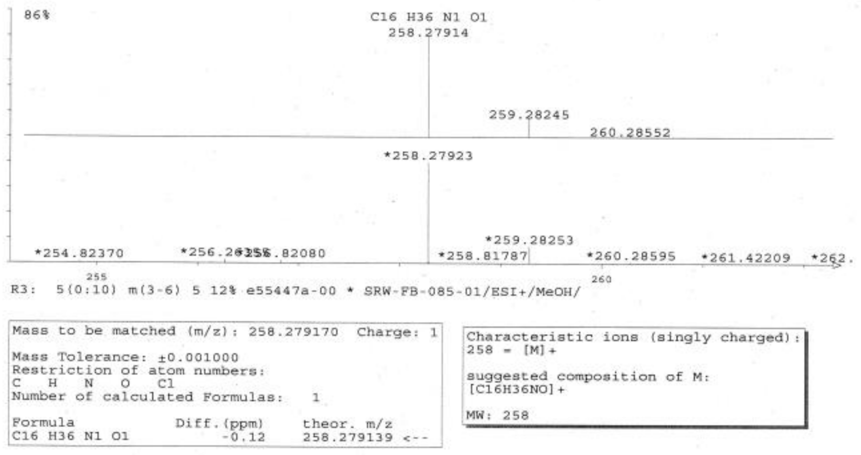

Figure S6: Mass spectra of 258,27914.

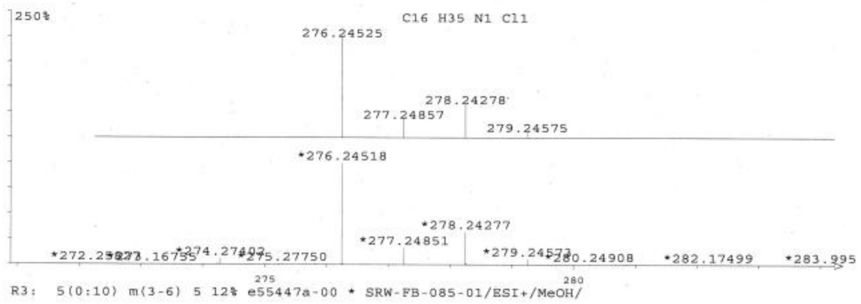

Mass to be matched (m/z): 276.245270 Charge: 1

Mass Tolerance: ±0.002000
Restriction of atom numbers:
C H N O Cl
Number of calculated Formulas: 3

Formula	Diff.(ppm)	theor. m/z
C16 H35 N1 Cl1	-0.07	276.245252 <--
C19 H32 O1	-1.83	276.244765
C17 H30 N3	-6.69	276.243421

Characteristic ions (singly charged):
276 = [M]+

suggested composition of M:
[C16H35NCl]+

MW: 276

Figure S7: Mass spectra of 276,24525.

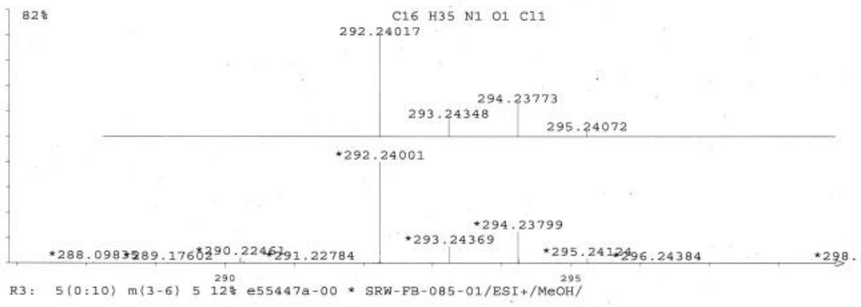

Mass to be matched (m/z): 292.240230 Charge: 1

Mass Tolerance: ±0.001000
Restriction of atom numbers:
C H N O Cl
Number of calculated Formulas: 2

Formula	Diff.(ppm)	theor. m/z
C16 H35 N1 O1 Cl1	-0.22	292.240167 <--
C19 H32 O2	-1.88	292.239680

Characteristic ions (singly charged):
292 = [M]+

suggested composition of M:
[C16H35NOCl]+

MW: 292

Figure S8: Mass spectra of 292,24017.

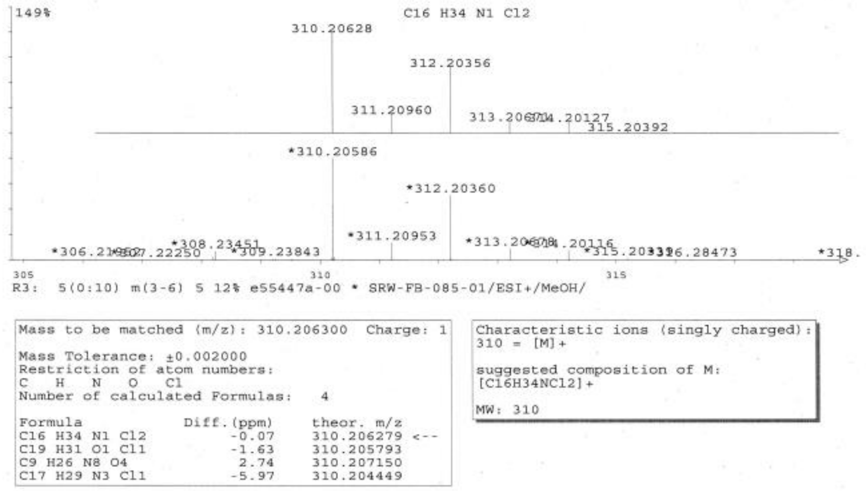

Figure S9: Mass spectra of 310.20628.

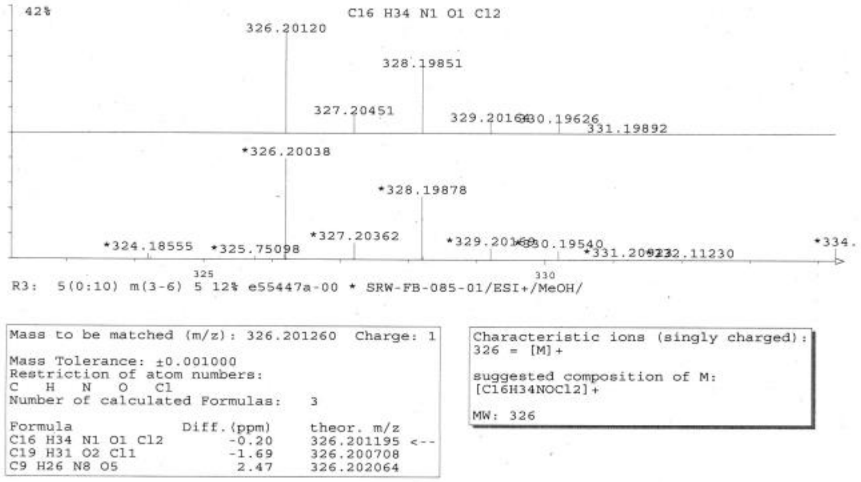

Figure S10: Mass spectra of 326,20120.

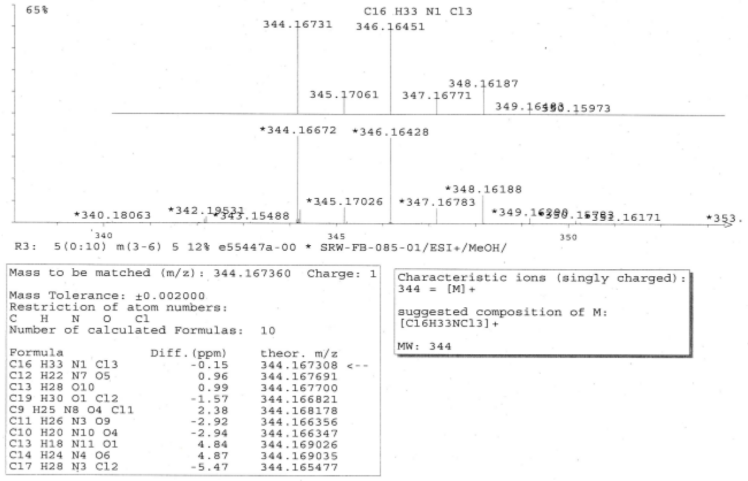

Figure S11: Mass spectra of 346,16451.

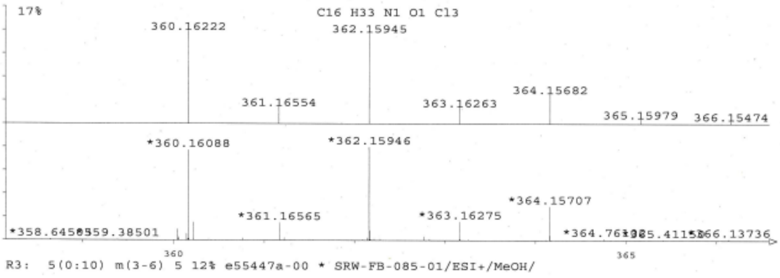

Mass to be matched (m/z): 360.162320 Charge: 1

Mass Tolerance: ±0.001000
Restriction of atom numbers:
C H N O Cl
Number of calculated Formulas: 6

Formula	Diff.(ppm)	theor. m/z
C16 H33 N1 O1 Cl3	-0.27	360.162223 <--
C26 H20 N2	-0.62	360.162097
C12 H22 N7 O6	0.79	360.162606
C13 H28 O11	0.82	360.162615
C19 H30 O2 Cl2	-1.62	360.161736
C9 H25 N8 O5 Cl1	2.14	360.163092

Characteristic ions (singly charged):
360 = [M]+

suggested composition of M:
[C16H33NOCl3]+

MW: 360

Figure S12: Mass spectra of 362,15945.

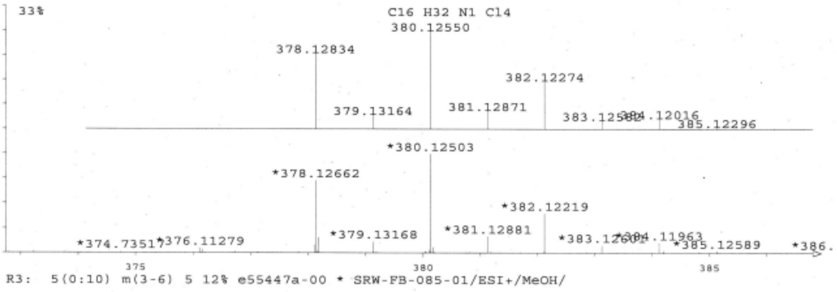

Mass to be matched (m/z): 378.128420 Charge: 1

Mass Tolerance: ±0.001000
Restriction of atom numbers:
C H N O Cl
Number of calculated Formulas: 8

Formula	Diff.(ppm)	theor. m/z
C16 H32 N1 Cl4	-0.22	378.128336 <--
C15 H18 N6 O6	-0.50	378.128232
C14 H12 N13 O1	-0.52	378.128223
C12 H21 N7 O5 Cl1	0.79	378.128719
C13 H27 O10 Cl1	0.81	378.128728
C19 H29 O1 Cl3	-1.51	378.127849
C29 H16 N1	-1.84	378.127724
C9 H24 N8 O4 Cl2	2.08	378.129206

Characteristic ions (singly charged):
378 = [M]+

suggested composition of M:
[C16H32NCl4]+

MW: 378

Figure S13: Mass spectra of 380,12550.

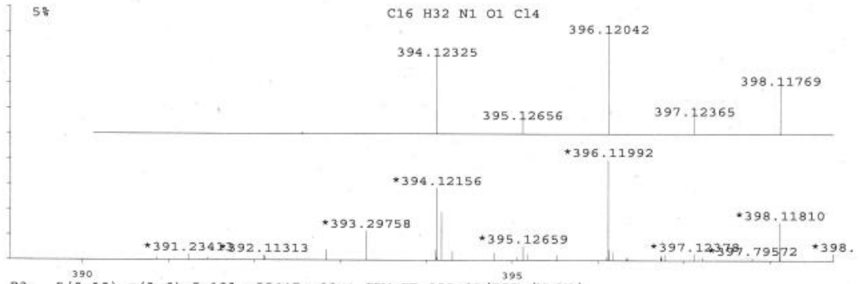

R3: 5(0:10) m(3-6) 5 12% e55447a-00 * SRW-FB-085-01/ESI+/MeOH/

Mass to be matched (m/z): 394.123290 Charge: 1

Mass Tolerance: ±0.001000
Restriction of atom numbers:
C H N O Cl
Number of calculated Formulas: 11

Formula	Diff.(ppm)	theor. m/z	
C16 H32 N1 O1 Cl4	-0.10	394.123251	<--
C15 H18 N6 O7	-0.36	394.123147	
C14 H12 N13 O2	-0.39	394.123138	
C26 H19 N2 Cl1	-0.42	394.123125	
C12 H21 N7 O6 Cl1	0.87	394.123634	
C13 H27 O11 Cl1	0.90	394.123643	
C19 H29 O2 Cl3	-1.33	394.122764	
C29 H16 N1 O1	-1.65	394.122639	
C9 H24 N8 O5 Cl2	2.11	394.124121	
C11 H25 N3 O10 Cl1	-2.51	394.122299	
C10 H19 N10 O5 Cl1	-2.54	394.122290	

Characteristic ions (singly charged):
394 = [M]+

suggested composition of M:
[C16H32NOCl4]+

MW: 394

Figure S14: Mass spectra of 394,12325.
